# Foraging niche segregation in Malaysian babblers (Family: Timaliidae)

**DOI:** 10.1371/journal.pone.0172836

**Published:** 2017-03-02

**Authors:** Mohammad Saiful Mansor, Rosli Ramli

**Affiliations:** 1 Institute of Biological Sciences, Faculty of Science, University of Malaya, Kuala Lumpur, Malaysia; 2 School of Environmental and Natural Resource Sciences, Faculty of Science and Technology, Universiti Kebangsaan Malaysia, Bangi, Selangor, Malaysia; Consejo Superior de Investigaciones Cientificas, SPAIN

## Abstract

Tropical rainforests are considered as hotspots for bird diversity, yet little is known about the system that upholds the coexistence of species. Differences in body size that are associated with foraging strategies and spatial distribution are believed to promote the coexistence of closely related species by reducing competition. However, the fact that many babbler species do not differ significantly in their morphology has challenged this view. We studied the foraging ecology of nine sympatric babbler species (i.e., *Pellorneum capistratum*, *P*. *bicolor*, *P*. *malaccense*, *Malacopteron cinereum*, *M*. *magnum*, *Stachyris nigriceps*, *S*. *nigricollis*, *S*. *maculata*, and *Cyanoderma erythropterum*) in the Krau Wildlife Reserve in Peninsular Malaysia. We investigated; i) how these babblers forage in the wild and use vegetation to obtain food, and ii) how these trophically similar species differ in spatial distribution and foraging tactics. Results indicated that most babblers foraged predominantly on aerial leaf litter and used gleaning manoeuvre in intermediate-density foliage but exhibited wide ranges of vertical strata usage, thus reducing interspecific competition. The principal component analysis indicated that two components, i.e., foraging height and substrate are important as mechanisms to allow the coexistence of sympatric babblers. The present findings revealed that these bird species have unique foraging niches that are distinct from each other, and this may apply to other insectivorous birds inhabiting tropical forests. This suggests that niche separation does occur among coexisting birds, thus following Gause’ law of competitive exclusion, which states two species occupying the same niche will not stably coexist.

## Introduction

The Malaysian tropical rainforest harbours a centre of biodiversity where many sympatric species coexist. Niche theory suggests that coexisting species will reduce the effects of interspecific competition through segregation of shared resources, which may result in phenotypic differences for the species in question [1]. However, the fact that many sympatric species do not differ significantly in their morphology to allow niche partitioning [2] has challenged this view. Understanding how different strategies have been used by sympatric species to utilise available resources can offer answers on how potential competitors coexist in the same habitat [3–4].

Resource segregation may be the consequence of long-term competitive adaptation over evolutionary time that defines how communities are structured [5–6]. As a result of such processes, different species may have different foraging strategies for resource exploitation. For instance, coexisting species frequently use different tactics that may result in capturing different prey [7]. This partitioning process is a significant evolutionary force in determining how competing species obtain their resources without causing competitive exclusion. Specialization has been linked to high foraging success resulting from the use of specific foraging tactics, perhaps in a consistent way over time [8], but this may limit foraging opportunities when the preferred resources are not present [9–10].

Generally, insectivorous birds in the tropical forest have high habitat specificity and are more confined to the forest interior than other avian feeding guilds [11]. Besides feeding specialization [12], insectivorous songbirds are also sensitive to microclimate changes [13]. Unlike fruits, flowers, and seeds, insects actively avoid birds, forcing insectivorous birds to develop numerous specialized niches and seek prey in preferred microhabitats [14]. Trophically similar species may use different foraging substrates and attack manoeuvres [15]. Numerous studies on foraging ecology of insectivorous birds have shown differences among similar bird species that inhabit the same habitat, which may explain coexistence [16–21]. However, foraging niche partitioning of closely related insectivorous birds is not well studied, especially for species from the same phylogenetic clade [22] within the same habitat, and particularly in Southeast Asia.

We focus on a diverse family of insectivorous birds, the Timaliidae, generally known as the babblers. Babblers are a major component of the tropical Asian avifauna, with a high level of sympatry. Babblers are one of the main groups of Malaysian insectivorous birds [23]. Most species of babblers are confined to the forest interior and have relatively limited distributions. Babblers are highly sedentary residents and are not strong flyers, foraging mostly in the understory [24]. They generally possess similar bill morphology (slender and either straight or slight decurved) and body size (ranging from 11 to 15 cm total length). The association between morphological traits and ecology is fairly well-known in birds, as the birds’ bill is a classic indicator of a trophic niche [25], and other biometric measurements, such as tarsus and wing length, can be linked to foraging attack manoeuvres, substrate use, and microhabitat preferences [26].

We studied the foraging ecology of nine babbler species that coexist in the central Peninsular Malaysia rainforest. These are black-capped babbler (*Pellorneum capistratum*), ferruginous babbler (*P*. *bicolor*), short-tailed babbler (*P*. *malaccense*), scaly-crowned babbler (*Malacopteron cinereum*), rufous-crowned babbler (*M*. *magnum*), grey-throated babbler (*Stachyris nigriceps*), black-throated babbler (*S*. *nigricollis*), chestnut-rumped babbler (*S*. *maculata*), and chestnut-winged babbler (*Cyanoderma erythropterum*). Following the hypothesis that similar species must vary in their requirements in order to coexist in the same habitat [27], we hypothesized that such species would have foraging niche segregation so that interspecific competition would be minimised, allowing coexistence. More specifically, our study addresses the following questions: (1) How do babblers forage in the wild and use vegetation to obtain food items? and (2) How do foraging strategies of these trophically similar species differ in terms of foraging height, foraging substrate, attack manoeuvre, and foliage density?

## Methods

### Study area

**Fig 1:**
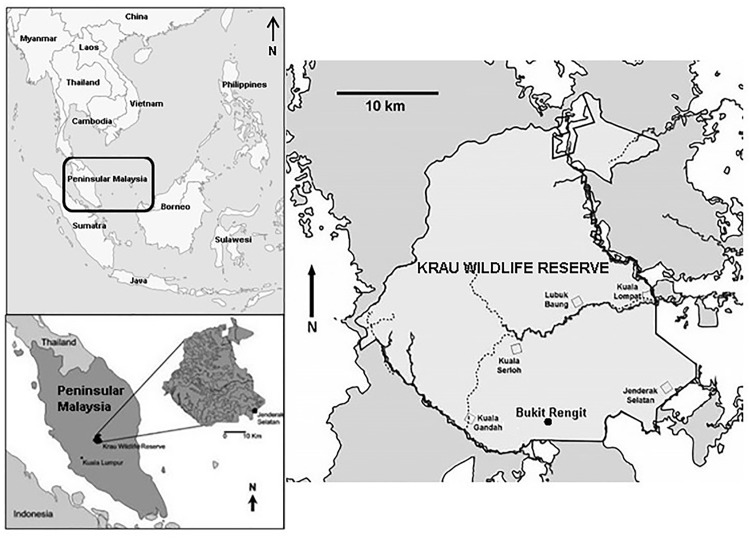
Map of Krau Wildlife Reserve, Pahang, Peninsular Malaysia. The reserve is represented by light grey, forest areas surrounding the reserve are indicated by dark grey, and non-forest areas are shown by white colour. Map adapted from [28].

The study was conducted in Bukit Rengit (3°35'40.02"N, 102°10'43.24"E), within the Krau Wildlife Reserve, a protected area located in Pahang, central Peninsular Malaysia (Fig 1; see [28]). The reserve consists of a large area of old-growth forest [29] and is the second largest protected area in Peninsular Malaysia after Taman Negara. It is approximately 624 km^2^, which ranges in elevation from 50 m (at Kuala Lompat) to over 2000 m (at the summit of Gunung Benom). The reserve is drained by three major river systems, i.e. Sungai Krau, Sungai Lompat, and Sungai Teris. The Krau landscape can be considered lowland or hill dipterocarp forests, with associated dominant tree species, including *Dipterocarpus cornutus*, *D*. *baudii*, *D*. *grandiflorus*, *Hopea sangal*, *Shorea acuminate*, *S*. *ovalis*, *S*. *leprosula*, *S*. *cutisii*, *Anisoptera laevis*, and *Vatica cuspidata* [30].

The daily temperature varies between a minimum of 23°C to a maximum of 33°C, and average rainfall is about 2000 mm, with maximum rainfall between September and December and between March and May, separated by two periods of minimum rainfall [30]. All five research stations in this reserve are managed and administered by the Department of Wildlife and National Parks. These stations are Kuala Lompat Research Station (KL), Lubuk Baung (LB), Kuala Sungai Serloh (KS), Kuala Gandah (KG), and Jenderak Selatan (JS). This study was conducted at Bukit Rengit near the Kuala Gandah station, from February 2014 to September 2015, a period which includes two breeding seasons and one migrating season.

### Foraging observations

Birds were located visually and randomly along six forest trails (S1 Fig), and followed opportunistically. Observations were performed for 10 days every month for a period of 20 months (February 2014–September 2015). Only one trail was sampled on any given day, so each trail was sampled for a total of 30 times over the whole study. To minimize repeated observation of the same individuals, conspecifics were only recorded when separated from each other by a distance of approximately 350–400 m or more. Birds were observed throughout the day, between 0730 and 1830 hours, but mostly in the early morning and late afternoon. Birds were observed as long as they could be kept in view, but only the initial (independent) foraging observations, i.e. first sighting of an individual bird, were used for statistical analysis to avoid problems with non-independent data. Observations for each foraging bird were made using 10 × 42 binoculars, recorded on voice recorder, and later transcribed into data spreadsheets. At least 30 independent observations were taken for each bird species to accurately represent the observed behavior [31–32].

The following data were recorded on each foraging bird encountered opportunistically: estimated height above the ground, foraging substrate, attack manoeuvre, and foliage density.

#### Foraging height

A foraging height is the level from which a food item is taken by the birds. Selected trees were marked as references for height standardization. This was estimated to 2 m interval, and grouped into four height categories (FH1: Ground/0 m; FH2: > 0–2 m; FH3: > 2–4 m; FH4: > 4–6 m; FH5: > 6–8 m; FH6: > 8–10 m).

#### Foraging substrate

A foraging substrate is the material (microhabitat) from which a food item is taken by the birds. These substrates include leaf surface, the underside of the leaf, branches, aerial leaf litter, and leaf litter.

#### Attack manoeuvre

The attack manoeuvre refers to how the food items are taken (attack) by the birds. This manoeuvre was categorised as follows: (i) glean—to pick food from a nearby substrate, reached without full extension of legs or neck; (ii) stretch—to completely extend the legs or neck to reach the food items; (iii) probe—to insert bill into softer substrate to capture hidden prey (iv) hang—to hang head down in order to reach food not obtainable from any other perched position; (v) hover—to maintain an airborne position by flapping wings and spreading the tail; and (vi) sally—to fly from a perch to attack a food item and then return to a perch. The terminology and strategy used to characterize attack manoeuvres follows [33].

#### Foliage density

This parameter was measured on a subjective scale from 1–5 in a 1-m diameter sphere around the bird. A series of numbers from 1 to 5 denoting the proportion of the area that was covered by the vegetation (leaves, bushes), ranging from 1 (covering less than 5% of the area) to 5 (covering more than 75% of the area), following a modified [34] cover abundance scale as described by [35–38].

### Statistical analyses

Principal component analysis (PCA) was performed to extract ‘patterns’ (i.e. linear combinations of raw variables that characterize foraging behavior) of bird species coexistence within each foraging niche partitioning category. PCA is a method that reduces data by forming linear combinations of variables and summarizes it into new synthetic variables (called principal components). Varimax rotation was used in order to facilitate axis interpretation. We used a scree analysis to determine the number of components with all foraging parameters in the analysis [39], and only axes with eigenvalues >1 were selected. From each of the principal components, we selected the high loading plots for the comparisons among birds, and these scores were used to interpret the foraging parameter gradients (i.e., vertical strata). PCA was performed using the Statistical Package for the Social Sciences [40], and the plotting was done using the Multivariate Statistical Package [41]. A hierarchical cluster analysis using correlation coefficients was used to group the species into distinctive guilds based on the frequency of all foraging parameters. This analysis was performed using the PAST software (PAleontological STatistics 2.17) [42].

## Results

A total of 354 independent observation bouts were made on the nine babbler species in the study area. More than 30 independent foraging observations, ranging from 30–64 observations were recorded for each studied species (see S1 and S2 Tables). Gleaning, without full extension of legs or neck to pick food from nearby substrate was frequently used in attack manoeuvres by the studied species and was exclusively used by *P*. *capistratum* and *P*. *malaccense* (S1 Table). Two species, *M*. *cinereum* and *C*. *erythropterum*, adapted a stretching manoeuvre by completely extending their legs or neck to reach the food items. *M*. *magnum* and *S*. *nigriceps* showed variation in the use of gleaning and stretching, while the foraging styles of *P*. *bicolor* were more varied (gleaning, stretching, and sallying).

Most of the birds foraged over a broad range of substrates but aerial leaf litter was the most frequently used substrate (*N* = 202 observations) and was commonly used by eight of the studied species except *M*. *cinereum* (S2 Table). Live green leaves were the second-most commonly used substrate (*N* = 121 observations) where the underside part (*N* = 72 observations) was preferred to the leaf surface (*N* = 49 observations).

All studied insectivore birds foraged at the understory level between ground level and 10 metres above the ground (S1 Table) where > 0–2 m stratum was used by most species, namely *P*. *bicolor*, *P*. *malaccense*, *S*. *nigriceps*, *S*. *nigricollis*, and *C*. *erythropterum*. The > 2–4 m of stratum was frequently used by *M*. *cinereum*, > 6–8 m of stratum was used by *S*. *maculata*, and ground level was predominantly used by *P*. *capistratum*. *M*. *magnum* exhibited variation in the use of foraging height (> 4–6 m and > 6–8 m).

Intermediate-density foliage (3 on this scale) was frequently used by most species (*M*. *cinereum*, *M*. *magnum*, *S*. *nigricollis*, *S*. *maculata*, and *C*. *erythropterum*) (S2 Table). Intermediate-highest foliage (4 on this scale) was frequently used by *S*. *nigriceps* and *P*. *bicolor*, while *P*. *malaccense* always used lowest-intermediate foliage (2 on this scale). *P*. *capistratum* showed variation in the use of foliage density (Scale 2 and Scale 3).

**Fig 2:**
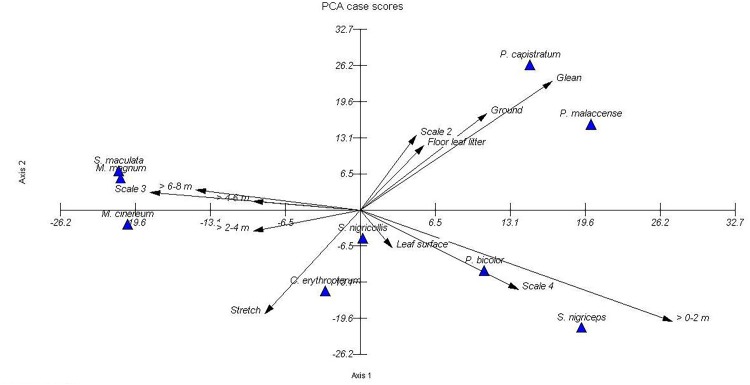
Distribution of nine babbler species based on Principal Component Analysis (PCA).

The principal component analysis (PCA) of nine babbler species yielded two components that explained 75% of the variation. The first principal component explained 56% of the data variation that was weighted on the foraging height parameter, whereas the second component explained 18% of the data variation that was weighted on the foraging substrate. The selected components were based on a scree plot curve and the range of percentage between components. In this case, the range of percentage between Dimension 2 (16%) and Dimension 3 (11%) was too small, thus only two instead of three components were selected for explanation. This analysis also identified 12 out of 22 relatively independent dimension classes, namely ground, > 0–2 m, > 2–4 m, > 4–6 m, > 6–8 m, leaf surface, floor leaf litter, glean, stretch, Scale 2, Scale 3, and Scale 4 of foliage density. Fig 2 illustrates the distribution of nine babbler species in the present study.

**Fig 3:**
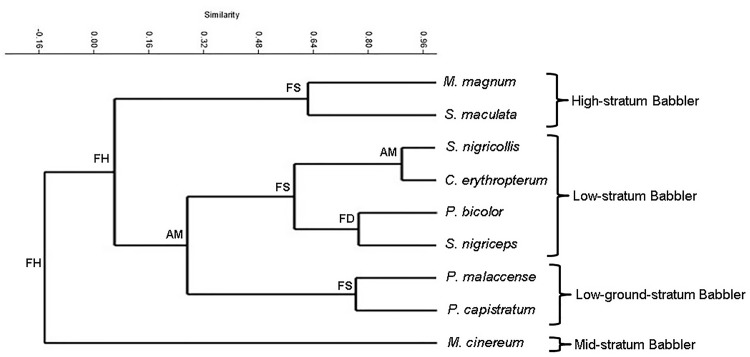
Interspecific relationships of nine babbler species, based on cluster analysis of foraging variables. FH = foraging height, AM = attack manoeuvres, FS = foraging substrate, FD = foliage density.

Niche segregation among bird species clearly explains the groups found by the cluster analysis (Fig 3). The analysis effectively divided nine babbler species into four main sub-guilds: (1) high-stratum babblers (i.e., *M*. *magnum*, and *S*. *maculata*), (2) mid-stratum babblers (i.e., *M*. *cinereum*), (3) low-stratum babblers (i.e., *S*. *nigricollis*, *C*. *erythropterum*, *P*. *bicolor*, and *S*. *nigriceps*), and (4) low-ground-stratum babblers (i.e., *P*. *malaccense*, and *P*. *capistratum*). Foraging height was important at the base of the cluster diagram and had divided foraging birds that used higher and lower vertical strata. For the high-glean insectivore group, the diagram separated *S*. *maculata* that primarily foraged on aerial leaf litter from those that vary in the use of foraging substrate (aerial dead leaves and live leaves). Furthermore, foraging substrate was important in the mid-glean insectivore groups to separate *P*. *malaccense* that mainly used aerial dead leaves from *P*. *capistratum* that is flexible to forage on either aerial dead leaves or ground leaf litter. At the terminal branches in the low-glean insectivore group, attack manoeuvre and foliage density were useful parameters for subdividing the foliage-preference groups into more specific groups.

## Discussion

We found that the babblers varied in their foraging niches (foraging height, substrate, foliage density, and attack manoeuvres) leading to a partitioning of resources. The degree of niche overlap among babblers differed for many species with respect to foraging parameters. By different foraging preferences, ecologically similar and closely related forest birds partition their resources [43–44].

Spatial segregation of babbler species demonstrates how they address the evolutionary trade-off between selecting food-rich microhabitats and optimum shelter with low predation risks from small mammals and reptiles. This trade-off is important for their life strategies and is linked with morphological adaptation [45]. This niche separation model assumes that resource partitioning results from different resource preferences and adaptations have resulted from past competition [46]. The outcomes of niche partitioning are consistent with several other studies of the foraging ecology of birds in the tropics [47–48] and temperate regions [49–50]. Although bird species richness in the tropical Malaysian rainforest is high, there is no evidence of competitive exclusion or strong competition shaping the bird species assemblage [51].

The factor analysis defined two principal components (i.e. foraging height and substrate) that are important as mechanisms to allow sympatric species coexistence, thus leading to the foraging guild of babbler assemblage. The first component is related to the use of vertical strata and can be interpreted as the primary resource partitioning among babblers, thus enabling their coexistence at a larger scale. This finding was consistent with the result of the cluster analysis, where foraging height was present at the base of the cluster diagram, thus highlighting the importance of this variable. Foraging height was important in subdividing the nine studied species into four foraging guilds, i.e. high-stratum babbler, mid-stratum babbler, low-stratum babbler, and low-ground-stratum babbler. This may have occurred over evolutionary time between related species; for example, the *S*. *maculata* foraged in higher strata than the *S*. *nigriceps*. The hypothesis that vertical distribution is a determinant of bird species assemblage was suggested by [52] and has been widely tested [53–55]. The findings of the present study provide further support that vertical distribution is important in niche partitioning of bird species and probably led to sympatric babbler species assemblages. In addition, variation in the carbon isotope δ^13^C of insects between the upper and lower vegetation [56] indicated differences in insect distribution, thus we assumed different prey were taken by height-partitioned birds. Different forest strata may support different groups and distributions of insects [57].

The niche segregation highlighted by the second component indicated that the substrate provides various foraging opportunities for the babblers. This could be interpreted as a secondary resource for the babbler assemblage, and this is associated with the vegetation structure. Although most babblers foraged on aerial leaf litter, they also fed on live green leaves, both on the surface and underside. A rich arthropod fauna in aerial dead leaves may hold great diversity of babbler species. From our preliminary results, this substrate supports many small coleopteran, hymenopteran, blattodea, and arachnids, thus providing many foraging opportunities for babblers. Aerial leaf litter suspended in the understorey plants may comprise significantly more arthropods and appeared to have unique species compared to live green leaves [58]. The abundance of this substrate that is suspended in many vertical strata of the understorey level allows the coexistence of many trophic dead-leaf foragers. At the same time it reduces competition and separates the foraging niche of dead-leaf foragers from green live-leaf foragers.

Higher frequency in the use of intermediate foliage density (3 on this scale) than dense foliage cover (covering more than 75% of the area) and open vegetation-cover suggests that the species tend to maximise their foraging opportunities while minimising predation risks. Easier arthropod detection in light penetrated areas [59] e.g. intermediate foliage cover was a likely explanation for why most babblers do not prefer dense vegetation cover. Foliage density may not be considered as important as other spatial dimensions but previous studies have revealed that the vegetation-cover structure along vertical placement distribution is important in influencing the foraging strategies of birds particularly those that mainly glean for prey items from certain foraging substrates [60–61]. In the current study, this parameter was also important in subdividing the foraging niche of *P*. *bicolor* from *S*. *nigriceps* (see Fig 3). This vegetation cover may also provide the best place to capture falling dead leaves which are the most preferred substrate of foraging babblers. Morphology of understorey plants plays an important role in capturing the falling dead leaves. The understorey shrubs, woody vines [62], ferns, small palms [63], and rattans [64], which were abundant in the study area are very useful in intercepting the forest canopy litterfall.

A recent study by [64] showed that *C*. *erythropterum* exhibited somewhat similar foraging strategies (e.g. substrate and attack manoeuvre) except they foraged at higher strata. Plasticity of foraging height among species may be driven by difference of habitat structure or the occurrence of mixed-species flocks [65–66]. Proximity to the forest edges may change the foraging opportunities for certain birds such as *C*. *erythropterum*, a highly disturbance-tolerant species. Certain species may forage more disproportionately at the forest edges than interiors possibly due to easier prey detection that has led birds to forage opportunistically [59]. For the latter [67] reported that the Shalley’s Greenbul (*Andropadus masukuensis)* foraged at a higher level when participating in mixed-species flocks (i.e. from mid-strata about 5–6 m above the ground to high-strata at about 13 m above the ground).

In addition, *C*. *erythropterum* seems to be the most generalist species, while *P*. *capistratum* is considered as the most specialist species. *C*. *erythropterum* is considered common and occurs across various habitat types, along the edge-interior gradient [68]. This large home range possibly reflects their preference for aerial leaf litter that is patchily distributed in the forest. The ground foraging position used by *P*. *capistratum* was less preferred by studied species, which makes them the most specialist. Specialist species with restricted ranges of resources are likely to be more sensitive to disturbance than generalist species [69]. Ground foragers are more vulnerable to predators than those foraging in higher strata and are found to have declined more in numbers than the arboreal foragers [55]. Near-to-ground foragers are usually exposed to ground predator (such as small mammals), arboreal predator (such as owls), or some reptiles (such as snakes), thus make them more vulnerable and lead to high disappearance. On contrary, higher strata or canopy birds may join large mixed-species flock to reduce predation risk.

The *S*. *nigricollis* and *C*. *erythropterum* seem to have the highest mean niche overlap, suggesting that they may forage at microhabitats offering similar cover and food resources. However, the use of attack manoeuvre was slightly different between these two species. The *S*. *nigricollis* usually uses gleaning, while the *C*. *erythropterum* prefers the stretching manoeuvre. Morphology differences (e.g. bill and body size) may be factors that result in these species having the highest mean niche overlap, thus allowing them to coexist in the same habitat. Variations in bill size possibly increase the range of food (prey group or size) that can be taken [70]. Birds display special morphological traits that relate to specialized attack manoeuvres that make foraging in certain microhabitats more efficient, undoubtedly helping to reduce interspecific competition [71–72].

The present data suggest that babbler species have a distinct and unique foraging niche which may apply to other insectivorous birds inhabiting lowland Malaysian rainforest. This spatial segregation is likely to be important to bird assemblages in most forest ecosystems. Vertical distribution which is related to a particular microhabitat extending along horizontal resources (foraging substrate and vegetation cover) and the attack manoeuvre seem to be the main factors in determining the foraging guild structure of babbler communities and their assemblages. We acknowledged the limitations that occur during observing unmarked small and shy passerine birds that may lead to repeated observation of same individual.

Although spatial distribution can affect habitat use among sympatric babbler species, further investigation is needed to define the significance of abiotic factors (e.g. light and temperature) and different habitat types. Changes in the use of these resources by birds in disturbed habitats have also not been well studied [13]. We expect that changes in habitat structure could lead to modifications in competitive dynamics among babblers [73]. In addition, other parameters such as plant species, dietary niche, and body size should be considered in future studies to reveal whether the forest birds are opportunists or are selective in using all resources [74–75]. A potential effect of abundance and availability of foraging substrates (e.g. aerial dead leaves) for forest insectivorous birds also requires further study. We expect that evaluation of the resource segregation of closely related species is only possible when several foraging dimensions are considered [76–77]. Although every parameter revealed small differences between species, the combination of several dimensions leads to a more complete assessment of how assemblages of the bird community are organized.

## Supporting information

S1 Fig Diagram showing six sampling trails at Bukit Rengit, southern part of Krau Wildlife Reserve, Pahang, Malaysia. Solid circle represents observation point.
(TIF)

S1 Table Foraging height and attack manoeuvre variables. Data are given as percentages (%).
(PDF)

S2 Table Foraging substrate and foliage density variables. Data are given as percentages (%).
(PDF)
