# Supplementary figures and images for: Foraging niche segregation in Malaysian babblers (Family: Timaliidae)
Source: PLoS One. 2017 Mar 2;12(3):e0172836. doi: 10.1371/journal.pone.0172836 (PMC5333830; doi:10.1371/journal.pone.0172836)

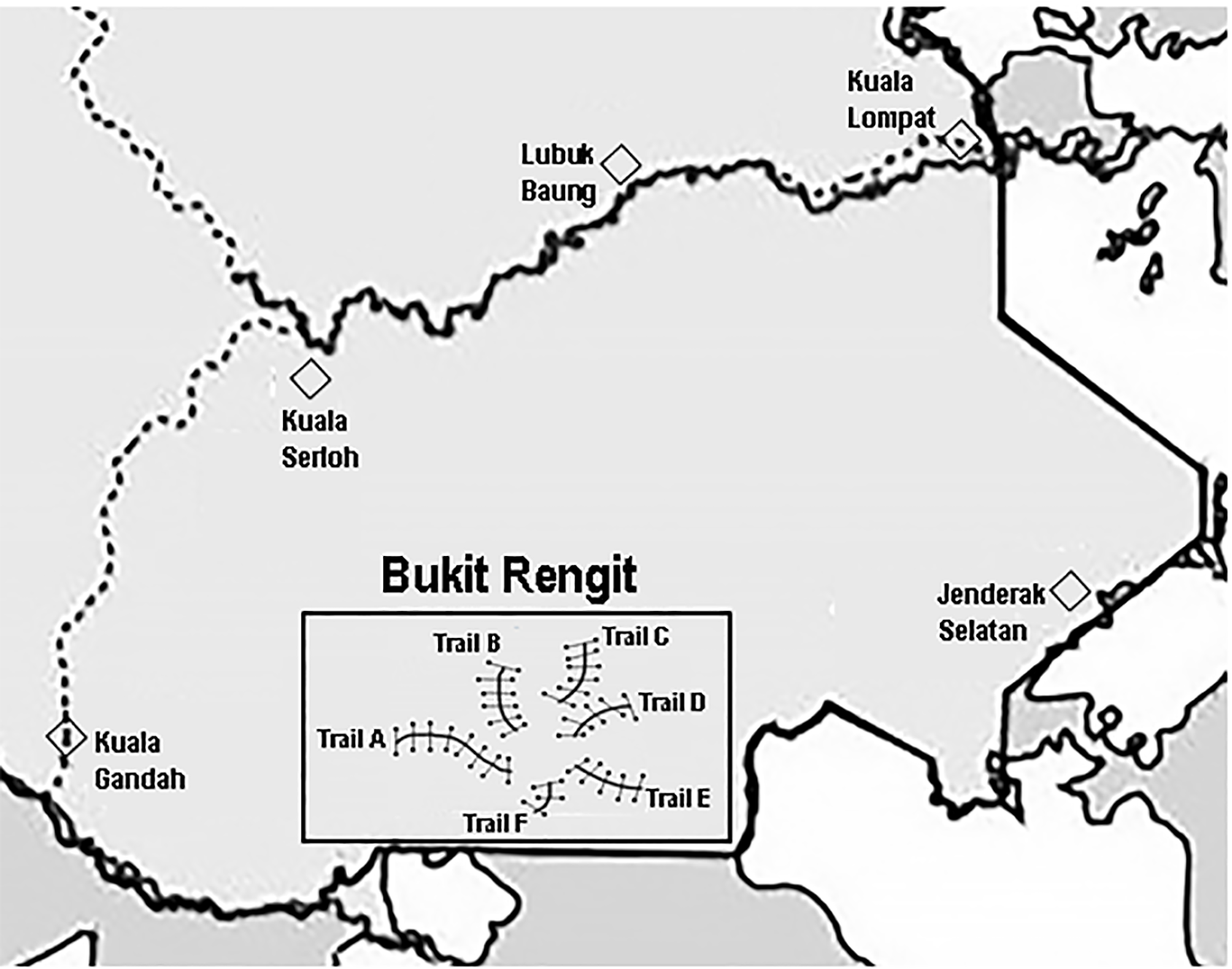

Supplement: S1 Fig — Solid circle represents observation point. (TIF) [file pone.0172836.s001.tif]
