# Supplementary material for: Foraging niche segregation in Malaysian babblers (Family: Timaliidae)
Source: PLoS One. 2017 Mar 2;12(3):e0172836. doi: 10.1371/journal.pone.0172836 (PMC5333830; doi:10.1371/journal.pone.0172836)
Supplement: S1 Table — Data are given as percentages (%). (PDF) [file pone.0172836.s002.pdf]

S1 Table. Foraging height and attack manoeuvre variables. Data are given as proportions (%).

Species	Foraging Height						Attack manoeuvre					
	**G**	**> 0-2**	**> 2-4**	**> 4-6**	**> 6-8**	**> 8-10**	**Glean**	**Stretch**	**Probe**	**Hang**	**Hover**	**Sally**
Pellorneum capistratum	55.56	44.44	0.00	0.00	0.00	0.00	100.00	0.00	0.00	0.00	0.00	0.00
P. bicolor	7.69	84.62	7.69	0.00	0.00	0.00	46.15	30.77	0.00	0.00	0.00	23.08
P. malaccense	33.33	66.67	0.00	0.00	0.00	0.00	100.00	0.00	0.00	0.00	0.00	0.00
Malacopteron cinereum	0.00	9.52	42.86	19.05	19.05	9.52	26.19	59.52	0.00	7.14	4.76	2.38
M. magnum	0.00	10.26	7.69	38.46	33.33	10.26	43.59	35.90	5.13	12.82	2.56	0.00
Stachyris nigriceps	0.00	100.00	0.00	0.00	0.00	0.00	44.44	44.44	0.00	11.11	0.00	0.00
S. nigricollis	0.00	75.68	24.32	0.00	0.00	0.00	62.16	29.73	8.11	0.00	0.00	0.00
S. maculata	0.00	16.13	16.13	6.45	45.16	16.13	45.16	12.90	25.81	16.13	0.00	0.00
Cyanoderma erythropterum	0.00	75.00	17.19	3.13	4.69	0.00	26.56	51.56	9.38	12.50	0.00	0.00
