# Supplementary material for: Foraging niche segregation in Malaysian babblers (Family: Timaliidae)
Source: PLoS One. 2017 Mar 2;12(3):e0172836. doi: 10.1371/journal.pone.0172836 (PMC5333830; doi:10.1371/journal.pone.0172836)
Supplement: S2 Table — Data are given as percentages (%). (PDF) [file pone.0172836.s003.pdf]

S2 Table. Foraging substrate and foliage density variables. Data are given as proportions (%).

Species	Foraging substrate					Foliage Density				
	Live green-leaf		Branch	Aerial leaf litter	Floor leaf litter	Scale 1	Scale 2	Scale 3	Scale 4	Scale 5
	Surface	Underside								
Pellorneum capistratum	0	11.11	0	44.44	44.44	11.11	33.33	33.33	22.22	0
P. bicolor	38.46	15.38	0	46.15	0	15.38	7.69	30.77	46.15	0
P. malaccense	16.67	26.67	0	50	6.67	0	50	16.67	33.33	0
Malacopteron cinereum	21.43	50	0	28.57	0	7.14	23.81	45.24	19.05	4.76
M. magnum	15.38	35.9	0	48.72	0	7.69	17.95	61.54	10.26	2.56
Stachyris nigriceps	22.22	33.33	0	44.44	0	0	0	0	77.78	22.22
S. nigricollis	0	16.22	8.11	75.68	0	0	0	70.27	29.73	0
S. maculata	0	0	32.26	67.74	0	16.13	0	67.74	16.13	0
Cyanoderma erythropterum	9.38	1.56	0	89.06	0	1.56	9.38	54.69	32.81	1.56
